# First photon-counting detector computed tomography in the living crocodile: a 3D-Imaging study with special reference to amphibious hearing 

**DOI:** 10.3389/fcell.2024.1471983

**Published:** 2024-10-23

**Authors:** Karl-Gunnar Melkersson, Hao Li, Helge Rask-Andersen

**Affiliations:** ^1^ Curator of Reptiles, Kolmårdens Tropicarium AB, Kolmården, Sweden; ^2^ Department of Surgical Sciences, Otorhinolaryngology and Head and Neck Surgery, Uppsala University, Uppsala, Sweden

**Keywords:** photon-counting computed tomography, μCT, crocodiles, hearing, underwater

## Abstract

**Background:**

Crocodiles are semi-aquatic animals well adapted to hear both on land and under water. Currently, there is limited information on how their amphibious hearing is accomplished. Here, we describe, for the first time, the ear anatomy in the living crocodile using photon-counting detector computed tomography (PCD-CT) and 3D rendering. We speculate on how crocodiles, despite their closed ear canals, can use tympanic hearing in water that also provides directional hearing.

**Material and Methods:**

A Cuban crocodile (*Crocodylus rhombifer)* underwent photon-counting detector computed tomography (PCD-CT), under anesthesia and spontaneous respiration. In addition two seven-month-old *C. rhombifer* and a juvenile Morelet´s crocodile (*Crocodylus moreletii)* underwent micro-computed tomography (µCT) and endoscopy. One adult Cuviérs dwarf caiman (*Paleosuchus palpebrosus)* was micro-dissected and video-recorded. Aeration, earflap, and middle ear morphology were evaluated and compared after 3D modeling.

**Results and Discussion:**

PCD-CT and µCT with 3D rendering and segmentation demonstrated the anatomy of the external and middle ears with high resolution in both living and expired crocodiles. Based on the findings and comparative examinations, we suggest that the superior earflap, by modulating the meatal recess together with local bone conduction, may implement tympanic hearing in submerged crocodiles, including directional hearing.

## Introduction

Crocodiles are semi-aquatic reptiles with a rich vocal repertoire, and their communication relies on their hearing and their ability to localize sound both in air and water. There have been several studies showing that crocodiles have exquisite hearing, which includes the ability to localize sound even while submerged. How this is accomplished is not entirely known (Manley, 1970; Klinke and Pause, 1980; Higgs et al., 2002; Bierman and Carr, 2005; Dinets, 2013; Young and Cramberg, 2024). Crocodiles may use bone conduction to detect and locate underwater sound, together with their well-developed cutaneous integumentary sensory organs (ISO-receptor system) to hear, sense, and locate water movements.

Sound in water may arise both as circular pressure variations and particle movements and as currents that are transmitted faster and with a longer range. In fish, particle motion is essential, and they may discriminate low frequencies, amplitudes, and time variations at ranges up to 800 Hz–1,000 Hz. As they are more sensitive to particle movements, they may also localize sound source (Bass and Ladich, 2008; Popper and Hawkins, 2019; Popper et al., 2019). By their similar density to water, there is no need for a middle and external ear, and sound is conveyed directly to the inner ear and swim bladder.

A direct path may prevail also in crocodiles whose heads are said to be transparent for sound (Higgs et al., 2002). Despite the lack of a swim bladder, crocodiles can hear frequencies up to 2,000 Hz in water (Fay, 1988; Manley et al., 2004) and possibly even higher frequencies. An argument against tympanic hearing under water in crocodiles and man is the finding that removal of air bubbles in the external ear canal do not influence auditory thresholds or bandwidth (Higgs et al., 2002). In man, the presence of air around the pinna and in the ear canal also seems not to improve underwater hearing sensitivity or sound localization confirming that bone conduction plays a more prominent role in human underwater hearing (Shupak et al., 2005). This could suggest that the middle ear is not used in underwater sound detection (Hollien and Brandi, 1969). It was concluded that crocodiles most probably hear through direct bone conduction via the skull bone. Cetaceans (whales and dolphins) can use acoustic cues to localize sound underwater, despite a retrogressive change of their external ears. This involves unique channeling to separate ultrasonic signals by spectral filtering and mechanisms to localize low frequency sound sources through inter-aural phase differences (Aroyan, 2001; Branstetter and Mercado, 2006). Snakes lack external and middle ears but may sense low-frequency vibrations through their middle ear bone located between the jaw and inner ear. Turtle and the small fully aquatic clawed frog (*Xenopus laevis*) use tympanic hearing to localize underwater sound despite their long wavelengths and fast speed that limit internal time and level differences between the ears (Christensen-Dalsgaard et al., 2012; Vedurmudi et al., 2018). Hence, several ingenious modifications have evolved among tetrapods, including internally coupled ears (ICEs), extracolumella shape, ear drums, and resonance phenomena, thus showing that tympanic hearing may be as effective under water as in air (Mason et al., 2009). Despite their similarity to the ears of birds, crocodiles’ ears seem to have adopted unique abilities to hear both in air and water.

The high velocity of sound pressure waves in water challenges auditory localization in amphibious animals. Given their inter-aural distance, crocodiles could exploit differences in the time of sound arrival and amplitude between the ears. They may also take advantage of a sound pressure gradient system through their ICEs to locate sound underwater (Rheinlaender et al., 1981; Vedurmudi et al., 2018). Studies in lizard and American alligator also demonstrated a muscular control of the tympanic membranes potentially modulating the ICE which may further increase sound localization in both air and water (Vedurmudi et al., 2016; 2020; Young and Cramberg, 2024).

How sound could enter their tympani under water regardless of their closed ears and provide directional hearing is not known. The superior earflap closes the ear and may protect the ear from mechanical damage and water pressure, and there is little evidence that it is associated with auditory stimulation (Shute and Bellairs, 1955). It contains both an elevator and a depressor muscle that raises and closes the earflap. These are connected to an acoustic plate that hinges beneath the superior squamosal bone. We aimed to analyze potential channels for sound to reach the middle ears also in closed ears. The superior earflap and meatal recess (external ear canal lateral to the tympanic membrane) including aeration were examined in a live spontaneously breathing crocodile using photon-counting detector computed tomography (PCD-CT) and compared with expired crocodiles investigated with µCT. In the live crocodile, the external ear canal is open, while in the expired fixed animal, it was closed, potentially representing a proxy for underwater conditions.

## Material and methods

### PCD-CT in living crocodile

One juvenile Cuban crocodile (*Crocodylus rhombifer)* with a weight of 1,020 g (18 months of age) was anaesthetized using an intramuscular (IM) injection of 5 mg/kg of Zoletil (tiletamin 25 mg/mL + zolazepam 25 mg/mL) in the right front leg that was repeated (after approximately 3 h) with 2 mg/kg in the left front leg. No antidote was given, and there was full mobilization following sleep. The crocodile maintained normal spontaneous breathing during the entire investigation. No unfamiliar muscular contractions or unusual motions of the earflaps were noticed. The crocodile was scanned at the CMIV research center at Linköping University Hospital, Sweden, using a photon-counting computer tomograph (Naeotom Alpha; Siemens Healthineers, Forchheim, Germany) with 0.2 mm sections with 50% overlapping with 10 sections/mm. The scanner provided increased separation of the irradiation spectrum into different energy levels, thus improving separation of different tissues. The advantages of PCD-CT over conventional CT include the spatial resolution that is fully comparable to Cone-Beam CT. PCD-CT technology detects radiation directly without converting X-ray beams into detected light. This largely avoids the detector electronic noise. Thereby a higher kernel/sharpness in the image than is possible with conventional CT including soft tissues can be obtained. The high resolution significantly reduces partial volume artefacts. The ability to selectively display individual energy levels (keV) from a single scan allows reconstruction of images at keV-level optimizing display of bone, soft tissues and reduce metal artefacts. Compared with Cone-Beam CT, PC-CT improves separation of tissue, allowing longer scanning periods and wider volumes reducing problems associated with Cone-Beam artefacts. Scan parameters for the whole-body were 120 kV; Care keV IQ Level 145; rotation time 0.5 s; 0.2 mm sections; 0.1 mm overlap; pitch 0.80; soft tissue kernel/sharpness Br48 and 1,024-pixel matrix. The head skeleton scan parameters were 120 kV; Care keV IQ Level 115; rotation time 0.5 s; 0.2 mm sections; 0.1 mm overlap; pitch 0.85; kernel/sharpness Hr84u; 1,024-pixel matrix. For bone kernel/sharpness Hr84u 1,024-pixel matrix. the head was scanned with Siemens default protocol for inner ear. To be sure of good image quality with no motion artifacts of the inner ear, the head was scanned twice. The second scan was taken from a standard human inner ear scan protocol. In retrospect, this was found unnecessary. The head images were reconstructed from the second scan and the whole body images were reconstructed from the first scan.

### Micro-computed tomography (µCT) and micro-dissections

Two juvenile males, seven-month-old and 2 years and eleven months-old *C. rhombifer* with a weight of 250 g and 1,050 g and a juvenile Morelet´s crocodile *C. moreletii* underwent micro-computed tomography (µCT) and 3D reconstructions. One adult Cuviérs dwarf caiman *Paleosuchus palpebrosus* was sexually mature and had a weight of 5,000 g. It was micro-dissected and the superior earflap and tympanic membranes were video-recorded after glutaraldehyde fixation and EDTA-decalification. They were anesthetized using Ketamine (5 mg) and medetomidine (0.05 mg) and euthanized using an intracardial injection of T-61 (0.4 mL). The skulls were separated, and the temporal bones were removed using an oscillating saw. The ears of the Cuban crocodile were immersed in 2.5% glutaraldehyde and 1% PFA in 2.5% phosphate buffer. Both ears of the *Crocodylus moreletii* were frozen, thawed and underwent micro-computed tomography (µCT) and 3D reconstruction without fixation. Aeration, earflap, and middle ear morphology were evaluated and compared after 3D modeling. The bones were scanned with µCT (SkyScan 1,176; Bruker, Kontich, Belgium) using the following parameters: source voltage 65 kV, current 385 µA, pixel size 9 μm, filter 1 mm Al, exposure time 1 s, frame averaging 2, and rotation step 0.30°. The projection images were acquired over an angular range of 360° with an angular step of 0.3°. In the resultant images, the image size was 4,000 × 2,672 pixels, and the pixel size was 9 µm. Projections were reconstructed using NRECON software version 1.7.0.4 (Bruker) based on the Feldkamp algorithm. A volume rendering technique was used to present a 2D projection of a 3D discretely sampled data set produced by the µCT scanner and visualized with the CTvox application (version 3.0; Bruker). Opacity and gray scale values were adjusted to create a realistic 3D view as close to that of the real bones as possible. Geometric measurements were performed, and pictures were taken with the 3D Slicer program (Slicer 4.6; www.slicer.org). The 3D Slicer is an open software platform for medical image informatics, image processing, and 3D visualization (Fedorov et al., 2012). The superior earflap and tympanic membrane were analyzed microscopically and video-recorded (Supplementary video).

### Segmentation and 3D visualization

The open-source program 3D Slicer (version 5.0.3, https://slicer.org) was used to perform segmentation and 3D visualization. Structures were segmented using automatic thresholding, island effects, and the scissor tool. Anatomically complex structures were manually segmented using the threshold painting tool. Scalar opacity mapping was adjusted, while 3D rendering the scans was performed to reveal structures of interest, for example, the structures beneath bones. Frontal serial sections were made across the meatal chamber and middle ear to fully assess the aeration and relationship to the earflaps.

### Histological processing

One temporal bone of the *C. rhombifer* and an adult (weight 5,000 g) Cuviérs dwarf caiman *(P. palpebrosus*) were placed in fixative for 48 h and in 0.1 M Na-EDTA for 3 weeks. The left superior earflap of the caiman crocodile was dissected and cut in smaller pieces for serial microscopy. The surrounding bone was further removed, and the ears were placed in 1% osmium tetroxide. The specimens were dehydrated in graded ethanol and embedded in Epon. The embedded specimens were mounted for semi-thin sectioning. Sections were stained in toluidine blue and photographed. Areas of interest were thin-sectioned, and the sections were stained in lead citrate and uranyl acetate and examined at 80 kV in a Tecnai™ G2 Spirit TEM (Thermo Fisher/FEI Company, Eindhoven, NL). Images were acquired with an ORIUS™ SC200 CCD camera (Gatan Inc., Pleasanton, CA, United States) using Gatan Digital Micrograph software.

## Results

### PCD-CT in the living *C. rhombifer* and tympanic aeration

**FIGURE 1:**
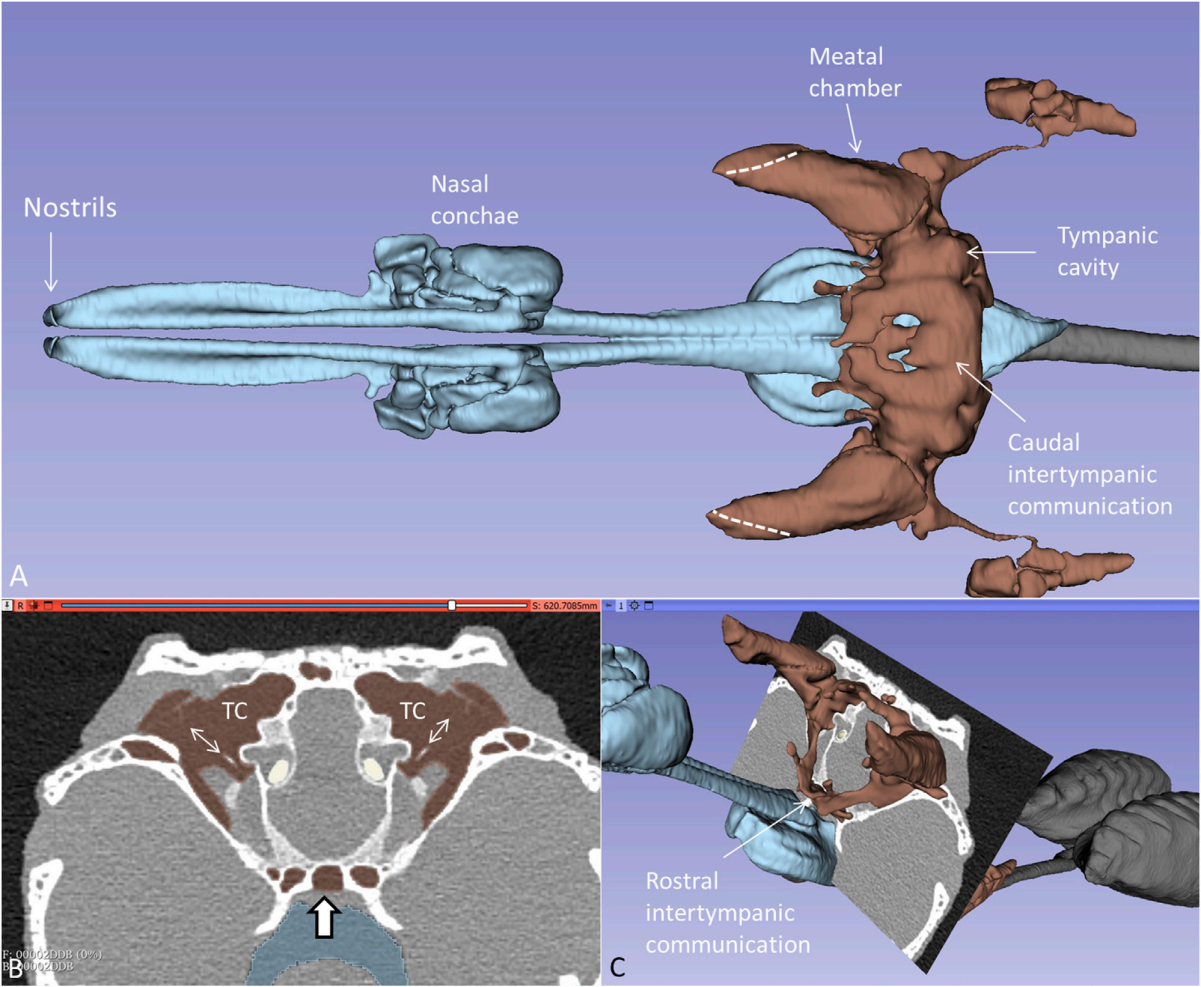
**(A)** Higher magnification of the segmented cranial air-filled spaces in the living *C. rhombifer*. The meatal chambers are fully aerated and open to the air anteriorly in a thin slit (broken lines). The ears are internally coupled caudally. **(B)** and **(C)** Frontal section at the posterior region of the tympanic cavity (TC) with aerated meatal recesses. Columellae and tympanic membranes are visible. The peritympanic air spaces continue into the pharynx (bold arrow) **(C)** Ears are also internally coupled rostrally.

PCD-CT and 3D renderings of the anaesthetized and spontaneously breathing juvenile *C. rhombifer* are shown in Supplementary Figure S1A–C. Different energy level and algorithms were used to show soft and skeletal tissue with high resolution. Larynx, vocal cords, trachea, and olfactory system were segmented separately (Supplementary Figure S1D, E). 3D volume rendering displayed the aerated tympanic cavities (TCs) connected to peritympanic cavities and the upper respiratory airway in the living crocodile. Lung bronchi could be resolved for analyses connected to unidirectional breathing (Schachner et al., 2013). Higher magnification of the segmented air-filled cranial spaces in the living Cuban crocodile is shown together with an orthogonal section in Figure 1A. The diameter of the tympanic membrane in the juvenile live crocodile was 10.3 mm. The tympanic air systems were interconnected both in an upper (caudal) and a lower (rostral) arrangement of air-filled cavities via median pharyngeal and pharyngotympanic recesses (Figures 1B, C). The tympanic membranes, columellae and extracolumellae could be resolved.

**FIGURE 2:**
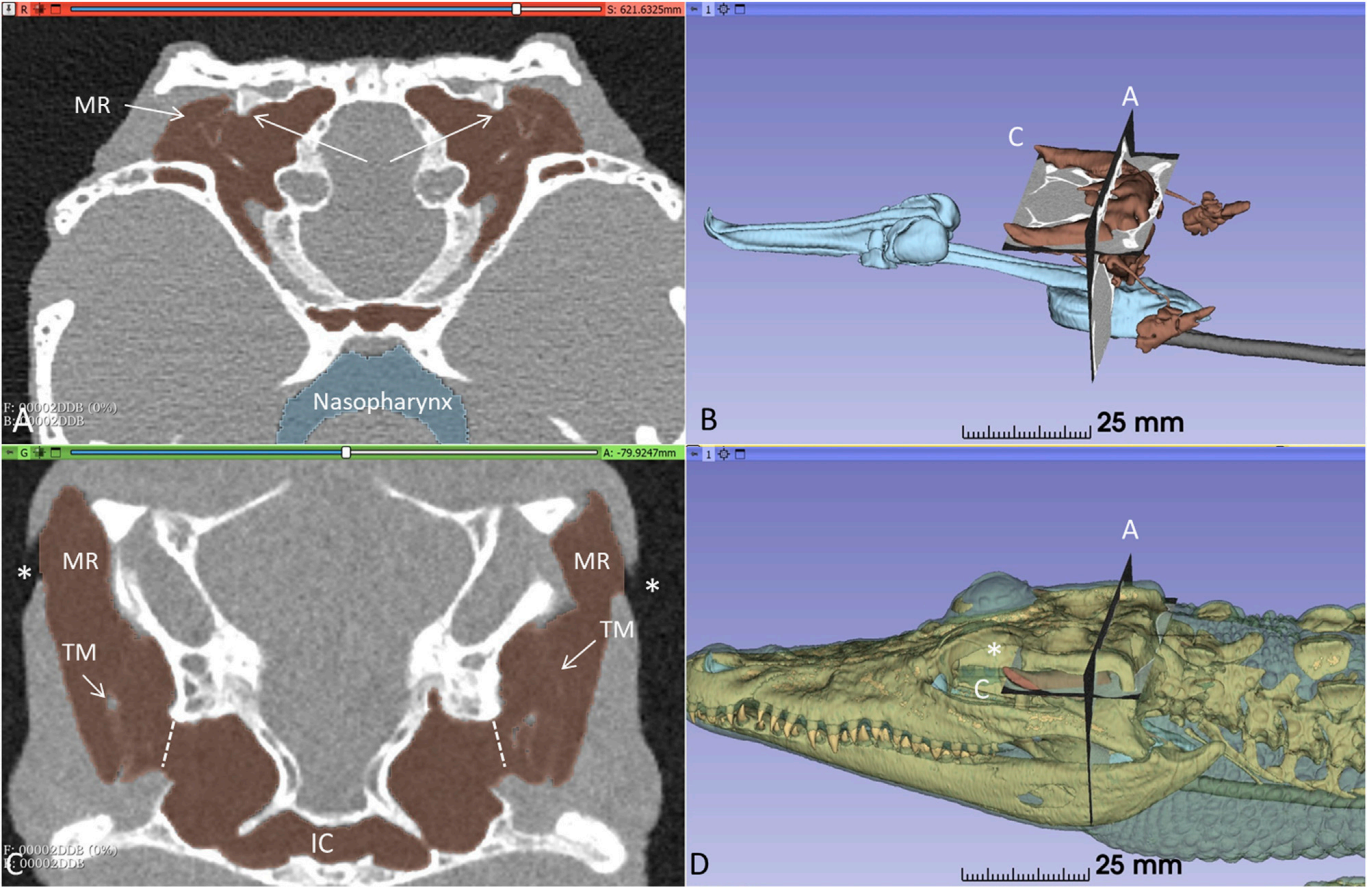
Orthogonal frontal **(A)** and horizontal **(C)** sections (A, C in figure **(B)** reveal internal coupling of the ears with caudally and rostral air-filled communications in the living crocodile. The extracolumellae are closely connected to the bony tympanic rings (arrows). The meatal recesses (MRs) are fully aerated in both ears and open anteriorly with a thin slit (*, **(C, D)**. TM: Tympanic membrane. IC; intertympanic communication.

Orthogonal frontal and horizontal sections at the level of the tympanic cavities are shown in Figure 2. The meatal recess (MR) is the aerated external meatus lateral to the tympanic membrane (Figure 2A), while the meatal chamber (MC) represents the bony ear canal margined medially by the bony tympanic plate reaching from the opening of the middle ear tympanon and subtympanic foramen. The MR opens anteriorly on the body surface with a thin slit to the surrounding air (Figures 2B–D). Both the MR and MC were fully aerated.

**FIGURE 3:**
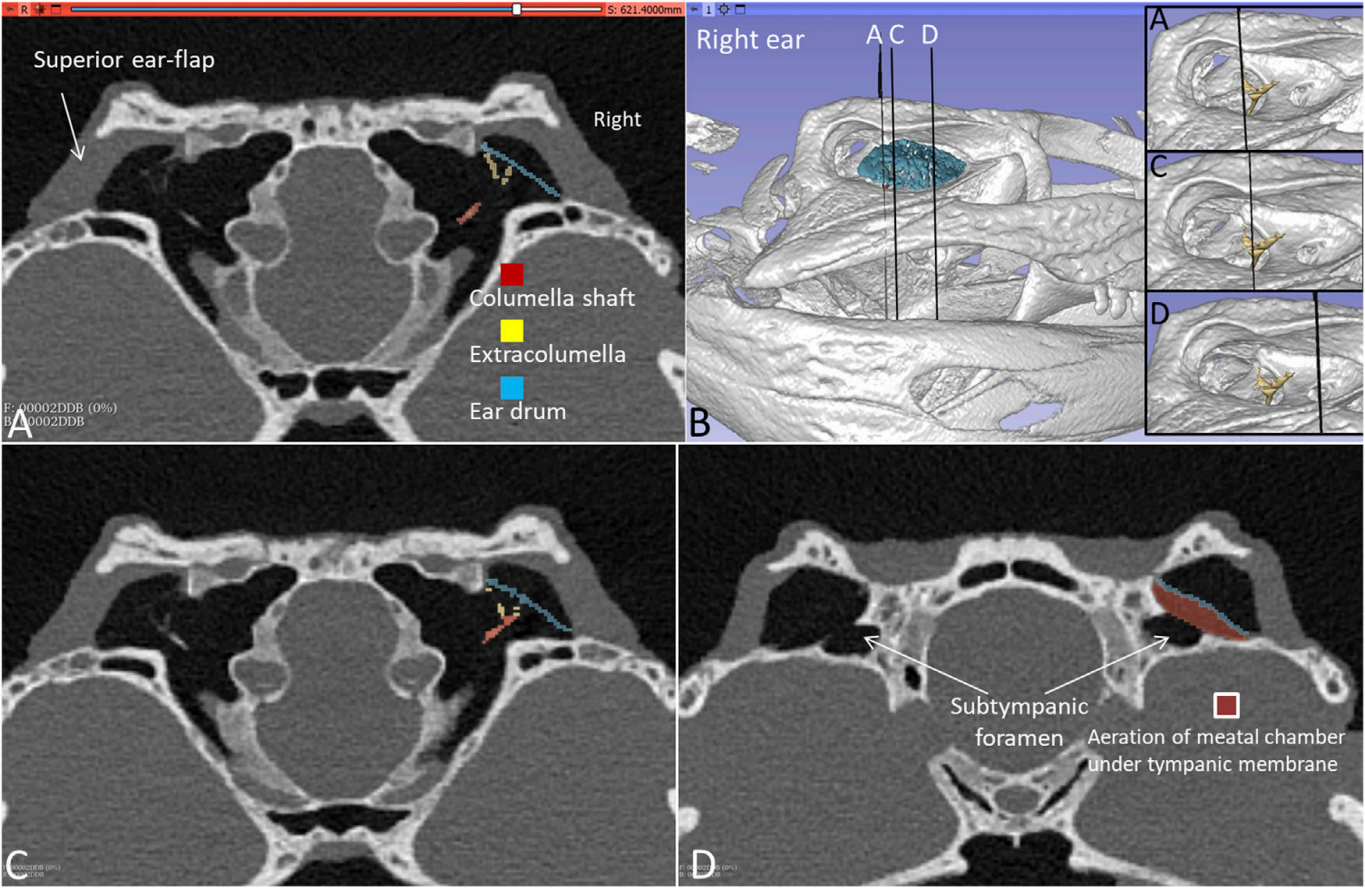
**(A)** PCD-CT with 3D rendering and segmentation demonstrating the aeration of the meatal recess in the living *C. rhombifer*. Section levels **(A,C,D)** of right ear are shown in **(B)** (insets without segmented ear drum). **(A)** Extracolumella touches the bony tympanic ring in right ear. The aeration of the meatal chamber underlying the tympanic membrane at the level of the subtympanic foramen is shown in **(D)**.

Figures 3A–D shows frontal sections and corresponding levels are seen at 3D rendering and segmentation of the tympanic membrane and columellae. TCs and MRs are fully aerated including the subtympanic spaces surrounding the subtympanic foramina (Figure 3D). The size of the meatal aerated space and variable distances between the tympanic membrane and earflap can be fully recognized along the MRs in Supplementary Figures S2, S3. The smallest distance was 0.55 mm at the posterior region (Supplementary Figure S2A) and the largest at the subtympanic foramen was 4.01 mm (Supplementary Figure S3H).

**FIGURE 4:**
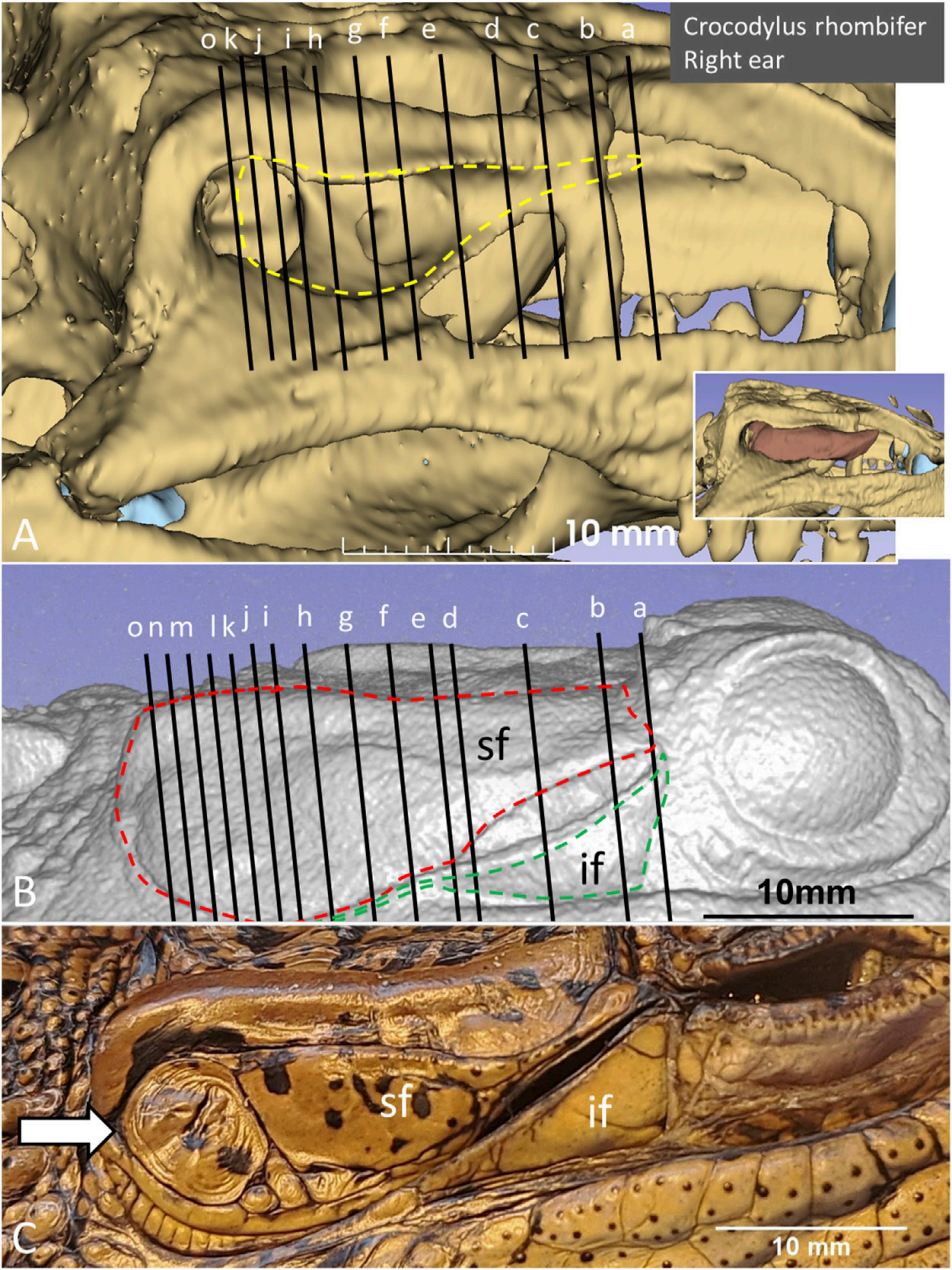
PCD-CT and 3D imaging algorithms of the right bony meatal chamber **(A)** and **(B)** skin with superior (sf) and inferior (if) earflaps in the living *C. rhombifer*. Inset shows ear canal air segmentation. Position of serial frontal sections are shown in bony and soft tissue algorithms and are displayed in Figure 6A. At area f-h, corresponding to the area of the subtympanic foramen, the sf bulges somewhat outwards. **(C)** A recently expired Cuban crocodile of similar size and age before aldehyde fixation. Its external ear canal is still somewhat open. Note the tympanic membrane-like texture at the posterior region of the superior earflap (white arrow). Its location corresponds to the location of the middle ear opening and extracolumella.

**FIGURE 5:**
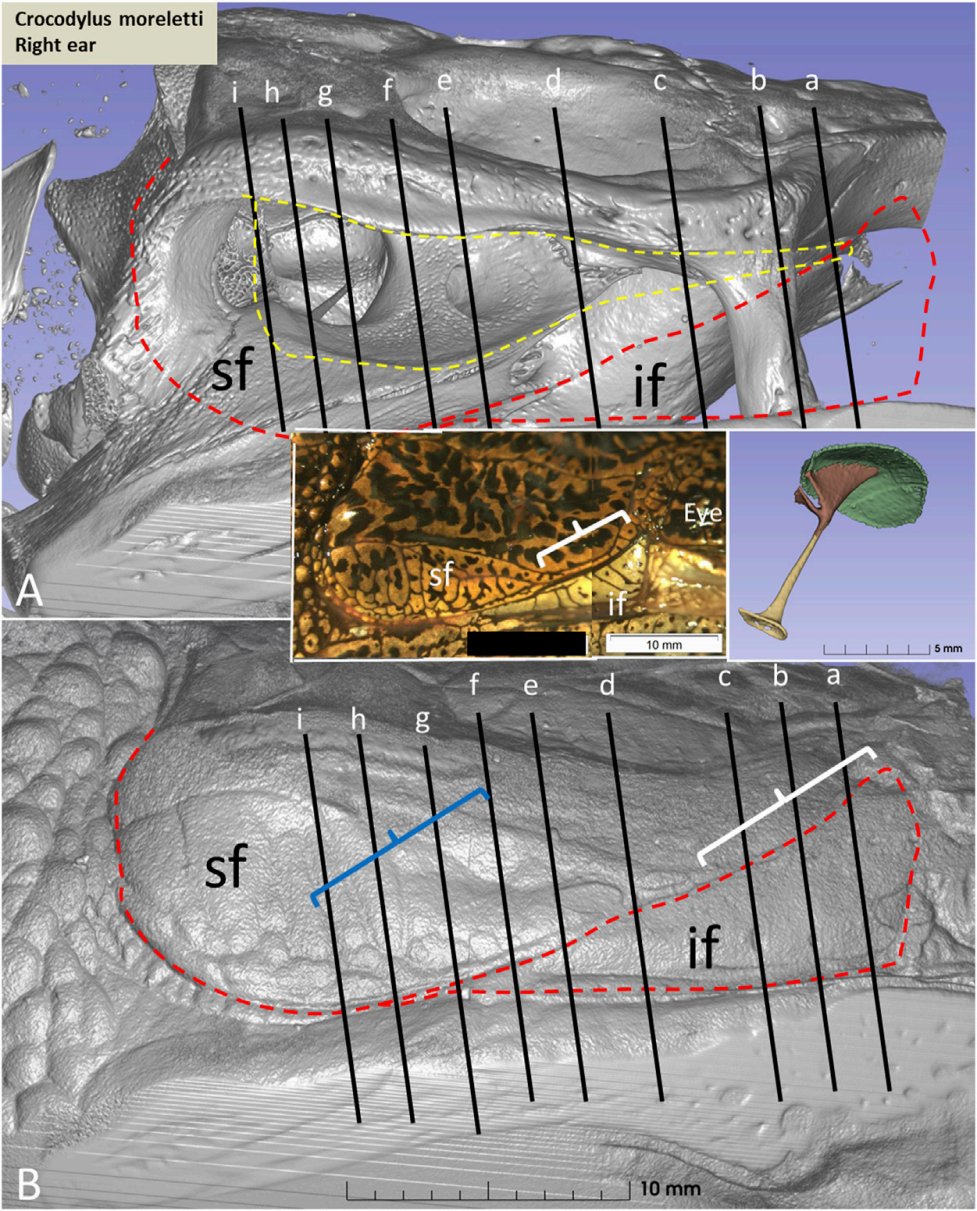
**(A)** µCT and 3D rendering of the meatal chamber of the right ear of the *C. moreletii*. **(B)**. Same projection and magnification including soft tissue structures. The corresponding µCT sections are shown in Figure 6B. Left inset shows photograph of the superior (sf) and inferior (if) earflaps including the closed external ear canal opening (white staple). Right inset shows columella and ear drum. Dashed yellow line in A shows the aerated space of the meatal recess and the red dashed line the peripheral margins of the upper and lower earflaps. At blue staple the size of the meatal recess was reduced.

**FIGURE 6:**
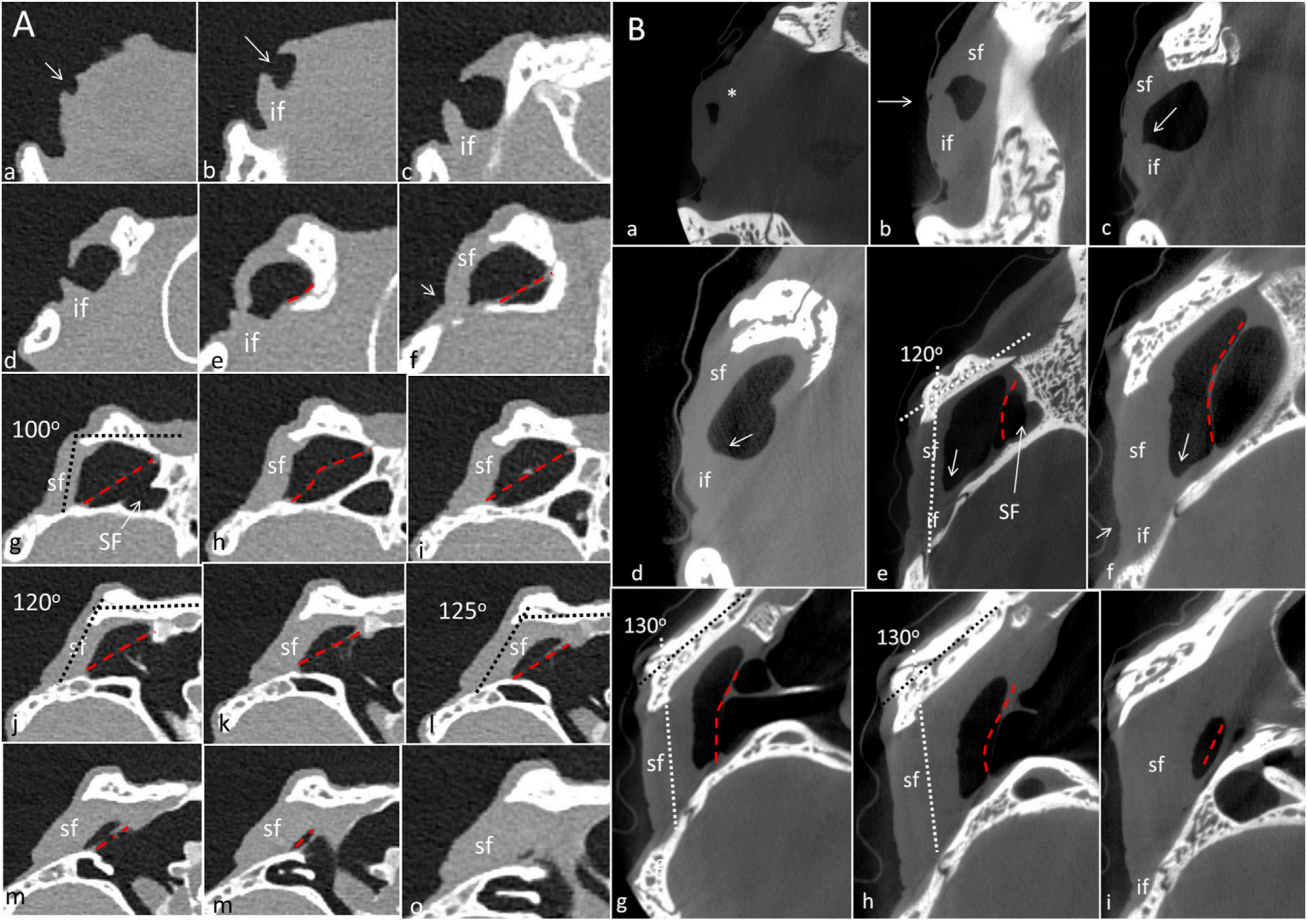
**(A)** PCD-CT serial frontal sections of the right meatal recess in the live crocodile shown in Figure 4. Anteriorly, the meatal recess is open to atmosphere (arrows). The inferior acoustic earflap (if) is tilted down- and outwards **(b–d)**. The recess closes at **(e)**. The superior earflap (sf) forms an almost perpendicular angle (100^o^) at the level of the subtympanic foramen (g, SF). At this region the recess volume is larger. It corresponds to the outpouching of the earflap seen in Figure 4B (level f and g). **(B)** Corresponding µCT sections and levels as demonstrated in *C. moreletii* shown in Figure 5. The meatal recess is completely closed and both earflaps have merged (white arrows). Red dashed lines; Tympanic membrane. SF; subtympanic foramen.*; Closed meatal recess behind the eye.

PCD-CT and 3D imaging algorithms of the right bony meatal chamber (A) and surface skin view (B) with superior and inferior earflaps in the living Cuban crocodile *(C. rhombifer)* are shown in Figures 4A, B. Inset in A shows segmentation of the aerated ear canal. Figure 4C shows the morphology of the earflaps in an unfixed, recently anaesthetized and expired (minutes) Cuban crocodile of similar size and age prior to fixation. Its external ear canal is open. There is a “tympanic membrane-like” structure at the posterior surface of the superior earflap that projects over the columella and middle ear opening. The serial frontal PCD-CT sections (a-o) are displayed in Figure 6A. An equivalent µCT and 3D rendering of the meatal chamber of the right ear in an expired Morelet´s crocodile (*Crocodylus moreletti)* is seen in Figures 5A, B. Corresponding µCT serial sections are shown in Figure 6B. The external ear canal is closed.

In the live crocodile serial frontal sections show that the MR is fully aerated and the external ear canal opens anteriorly in a 3.6 × 6.7 mm large slit behind the eye (Figure 6A). At f-h, corresponding to the area of the subtympanic foramen, the superior earflap bulges somewhat outwards. The anterior lip of the inferior earflap is displaced inferiorly with outward rotation, assumingly by muscular contraction (plate d and e in Figure 6A). There is also a slight upward retraction of the anterior-inferior lip of the superior earflap (sf). In the expired crocodile the MR and earflaps are entirely closed (Figure 6B). The volume of the MR seem to differ in the living and non-living crocodiles as well as the vertical angles of the superior earflap. This is most pronounced at the level of the subtympanic foramina. The tympanic membrane in the expired crocodile seems to lateralize into the MR while the superior earflap appears somewhat medialized. Conversely, in the live crocodile the superior earflap bulges outward with dilated MR particularly at the region of the subtympanic foramina. In the expired crocodile the MR is aerated but the distance between the superior earflap and tympanic membrane appears reduced and is at umbo 0.64 mm (Supplementary Figure S4).

**FIGURE 7:**
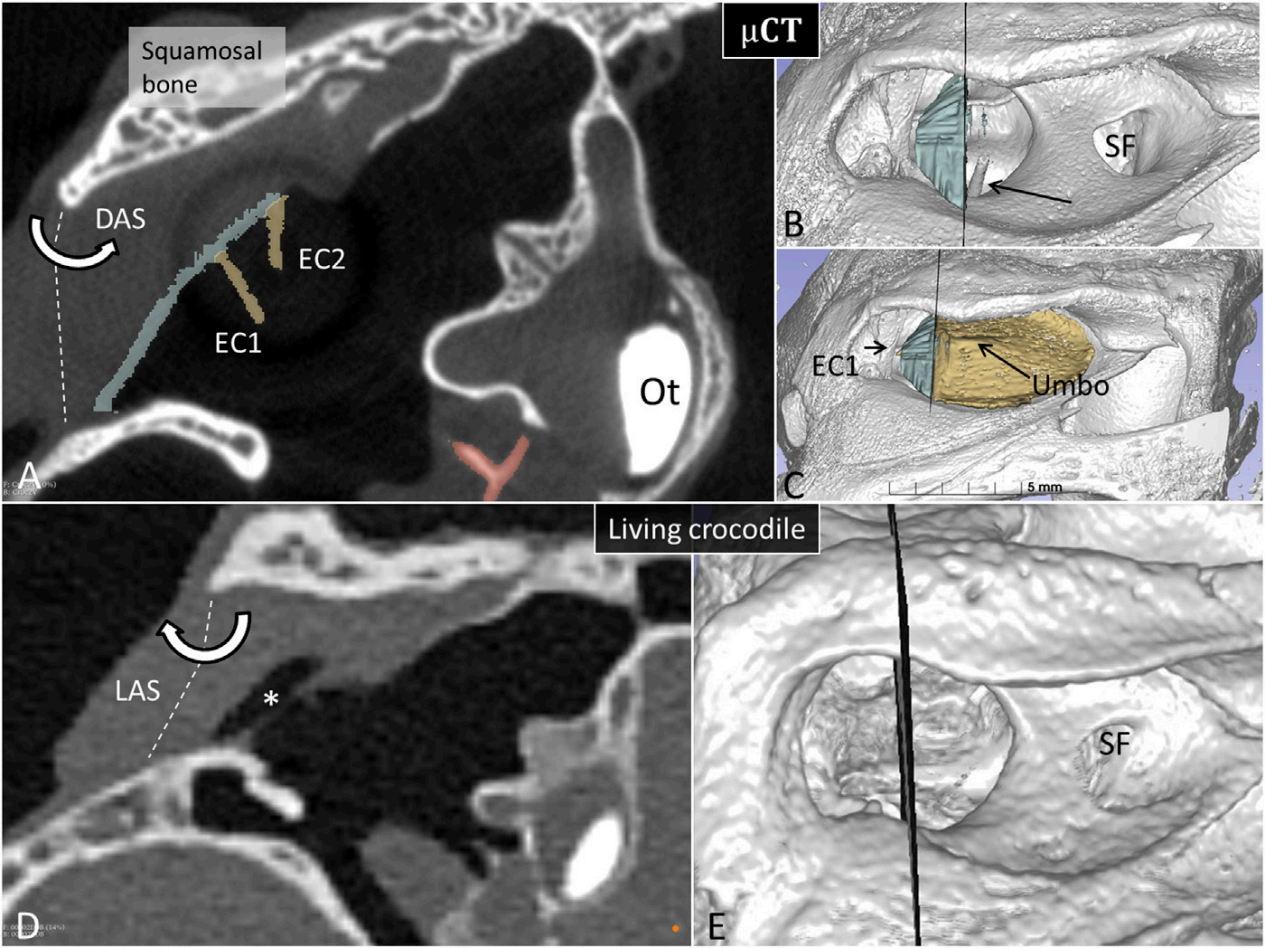
Matched µCT and PCD-CT sections of the right meatal recess in the expired **(A)** and live *C. rhombifer* **(D)**. Corresponding levels are shown at 3D rendering in **(B,C,E)** respectively. The posterior region of the meatal recess in A contains no air while in D it is aerated (*). **(B)** The posterior portion of the tympanic membrane that adheres to the superior earflap is segmented and marked in blue. Arrow shows columellar shaft. SF, subtympanic foramen. **(C)** The anterior aerated portion of the tympanic membrane is marked in yellow. Extracolumella EC1 and EC2 are yellow. The levator (LAS) and depressor (DAS) muscles attach to the acoustic plate within the superior earflap that act cranially as a rotating hinge. Ot, otolith.

**FIGURE 8:**
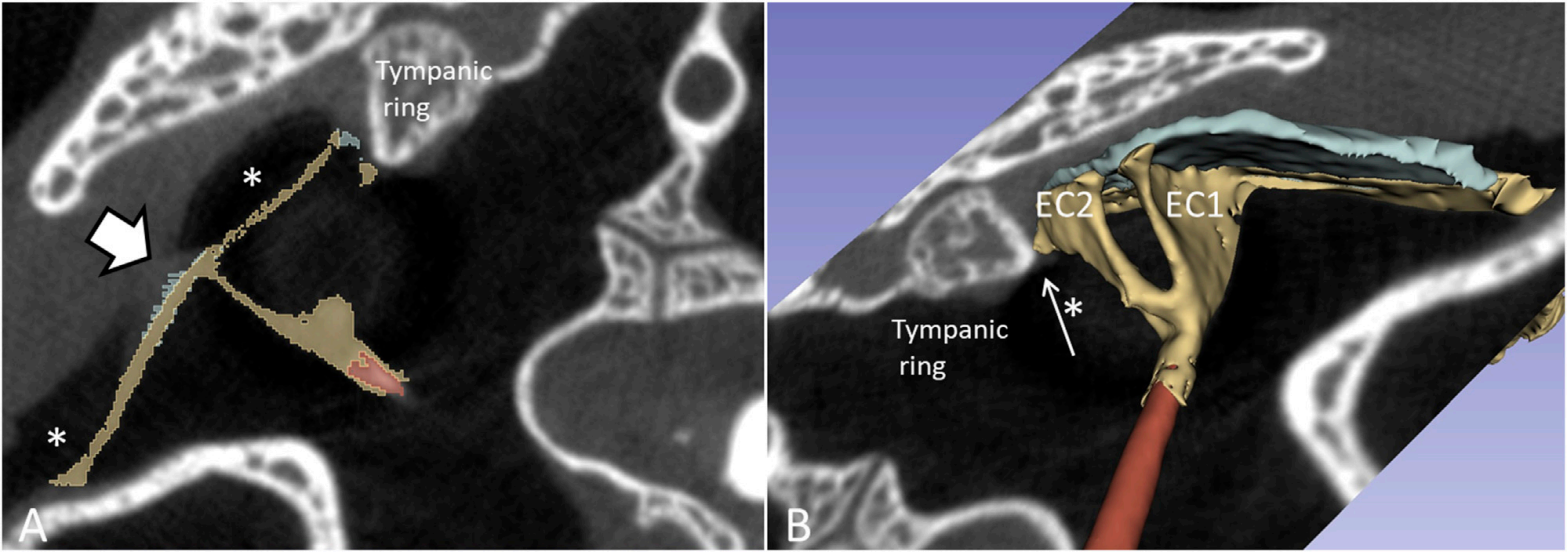
µCT **(A)** and 3D segmentation **(B)** of the middle ear at the level of the umbo. The meatal recess is partly aerated. The extracolumella adheres to the bony tympanic ring in **(B)** (*).

To exclude the possibility that fluid in the MR explained diminished or lack of aeration, the recess was gently opened in one Cuban crocodile followed by µCT investigation. Figure 7 shows µCT and PCD-CT orthogonal sections demonstrating different aeration of the MR in the live and expired Cuban crocodile (*C. rhombifer)* at comparable regions of the tympanic membrane. Frontal sections and 3D renderings in the terminated Cuban crocodile show there is no aerated space between the superior earflap and the posterior region of the tympanic membrane (marked blue) while corresponding PCD-CT section in the living crocodile show a fully aerated MR and the superior earflap is completely separated from the tympanic membrane. In the expired Cuban crocodile the medial surface of the superior earflap reaches the posterior region of the tympanic membrane and umbo, partly surrounded by air (Figure 8, Supplementary Figure S5). Combined µCT and 3D rendering demonstrated the columella, extracolumella and their topographic relationship to the tympanic bony ring.

### Microdissection and video-recording of the meatal recess and superior earflap

**FIGURE 9:**
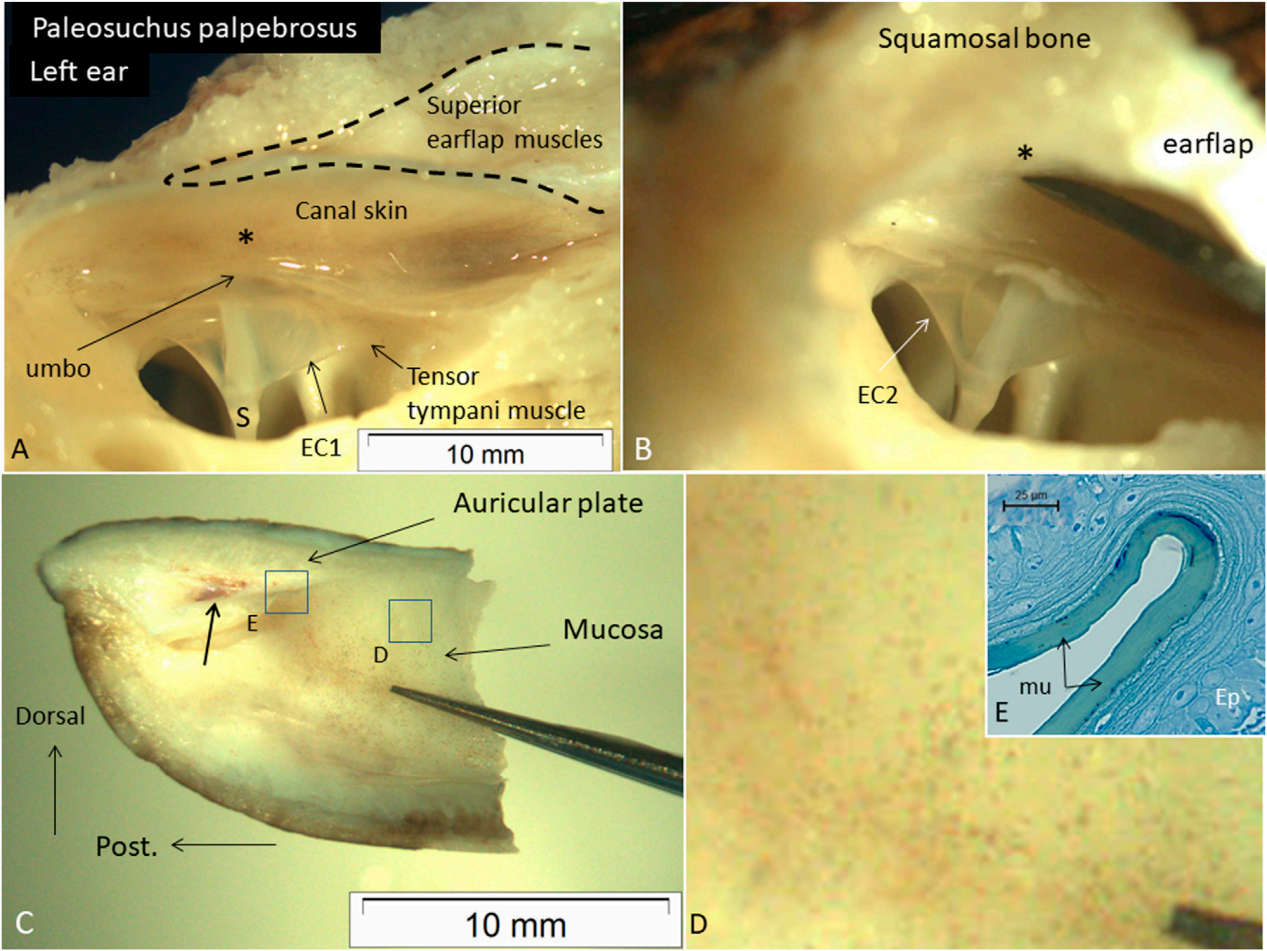
**(A)** Dissected left ear in an expired adult Cuviérs dwarf caiman (weight 5,000 g) (*P. palpebrosus*) showing tympanic membrane and the narrow posterior region of the meatal recess and extracolumella (EC1). There is a short distance between the posterior ear canal skin and umbo (*). The tensor tympani muscle reaches the EC1. **(B)** The canal wall and somewhat bulging muscles are retracted from the tympanic membrane. **(C)** Posterior part of the superior earflap (medial view). Muscle attachment to the auricular plate (arrow). Framed areas are magnified in D and **(E)**. **(D)** Higher magnification of framed area in C exhibits several micro-pigmentations. **(E)** Medial surface of the superior earflap with a smooth mucosa (mu) surrounded by concentric cell arrangements. Ep; epithelium. Scale bar is 25 µm. Glutaraldehyde fixation and toluidine blue staining. S: shaft of the columella. EC2; extracolumella 2.

Microdissection of a Cuvier´s dwarf caiman (*P. palpebrosus*) also demonstrated the close relationship between the posterior region of the tympanic membrane, the umbo, and the posterior canal wall. The tensor tympani muscle reached the tympanic annulus and extracolumella. The superior earflap muscles occupied the epitympanic space and faced the posterior-superior canal wall and posterior region of the MR (Figures 9A, B). There was no fluid obliterating the posterior region of the MR. Manual compression of the posterior-superior canal wall displaced the superior earflap muscles and decreased the distance to the umbo. The medial surface and insertion of the superior earflap muscles at the posterior region of the auricular plate are shown in Figures 9C, D. Histological analysis shows that the medial surface of the superior earflap is structurally modified. A loose arrangement of cellular specializations form concentric “strings” interconnected between the epithelial cell layer and the superficial extra-cellular mucosal layer (Figure 9E).

### µCT and 3D rendering in expired crocodiles

**FIGURE 10:**
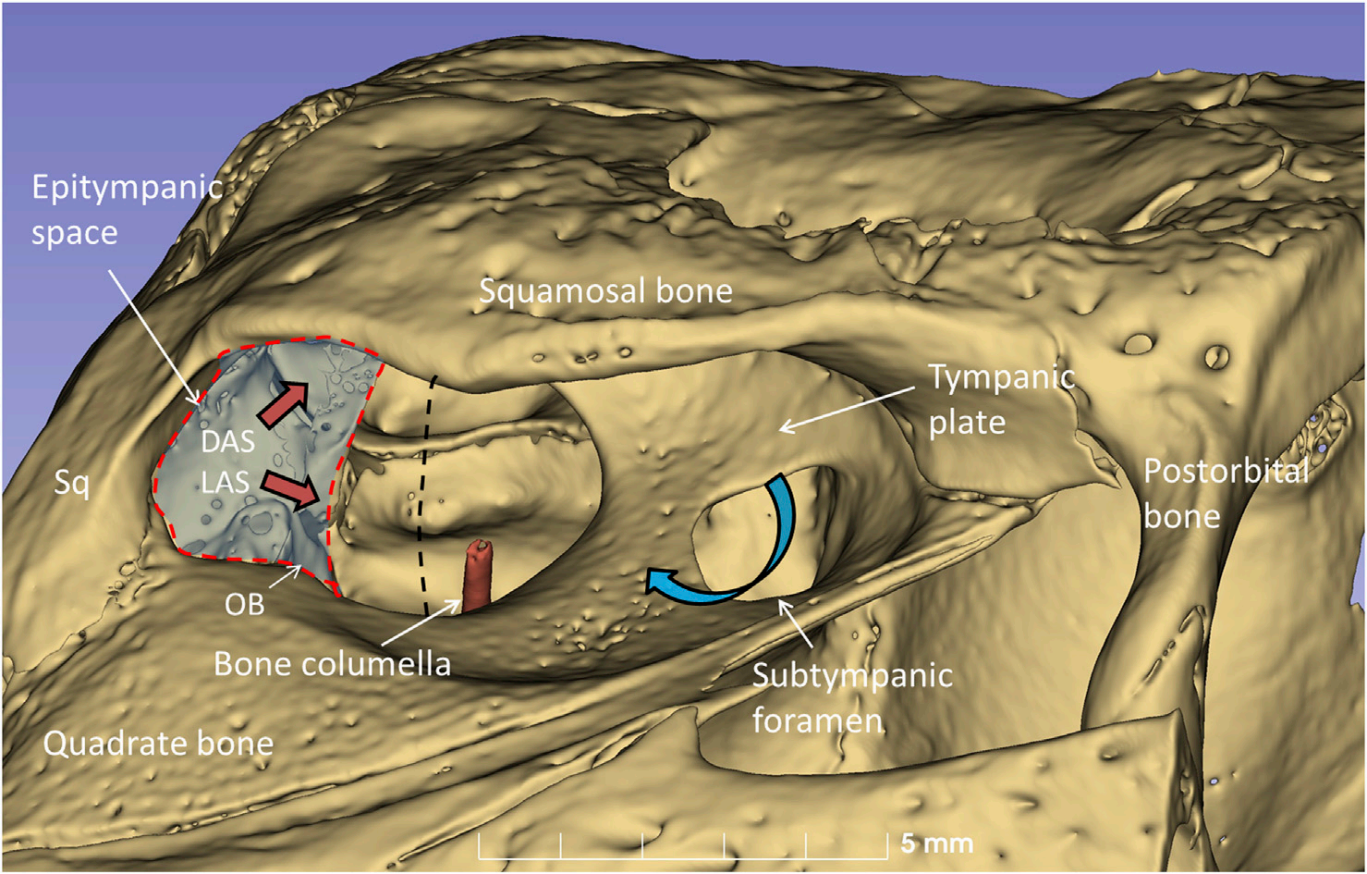
µCT-3D rendering of the right bony external ear in a seven-month-old *C. rhombifer* - lateral view. There are two foramina and communication pathways between the meatal chamber and middle ear: one larger that houses the columellar shaft (red) and one smaller one, the subtympanic foramen, that is located anteriorly and aerates the anterior ear drum (blue arrow). A posterior epitympanic space (red interrupted line) contains muscles connected to the tympanic membrane and extracolumella (tensor and stapedius muscles) and the levator and depressor auricular superior muscles (LAS, DAS, red arrows). LAS and DAS muscles are attached to the acoustic plate. The superior earflap is attached to the lateral boundaries of the squamosal and postorbital bones. The dashed dark line indicates the margin between anterior and posterior portions of the tympanic membrane shown in Figure 11.

µCT and 3D rendering of the crocodile ears showed even higher resolution. The diameter of the tympanic membrane in the seven-months-old Cuban crocodile (*C. rhombifer*) was 7.6 mm. The architecture of the bony meatal chamber and the openings into the middle ear with bony columellae, subtympanic foramina and epitympanic space are shown in Figure 10. The posterior space (here named the epitympanic space) contains muscles connected to the tympanic membrane and extracolumella and the levator and depressor auricular superior muscles (LAS, DAS). The superior earflap is attached to the lateral boundaries of the squamosal and postorbital bones.

**FIGURE 11:**
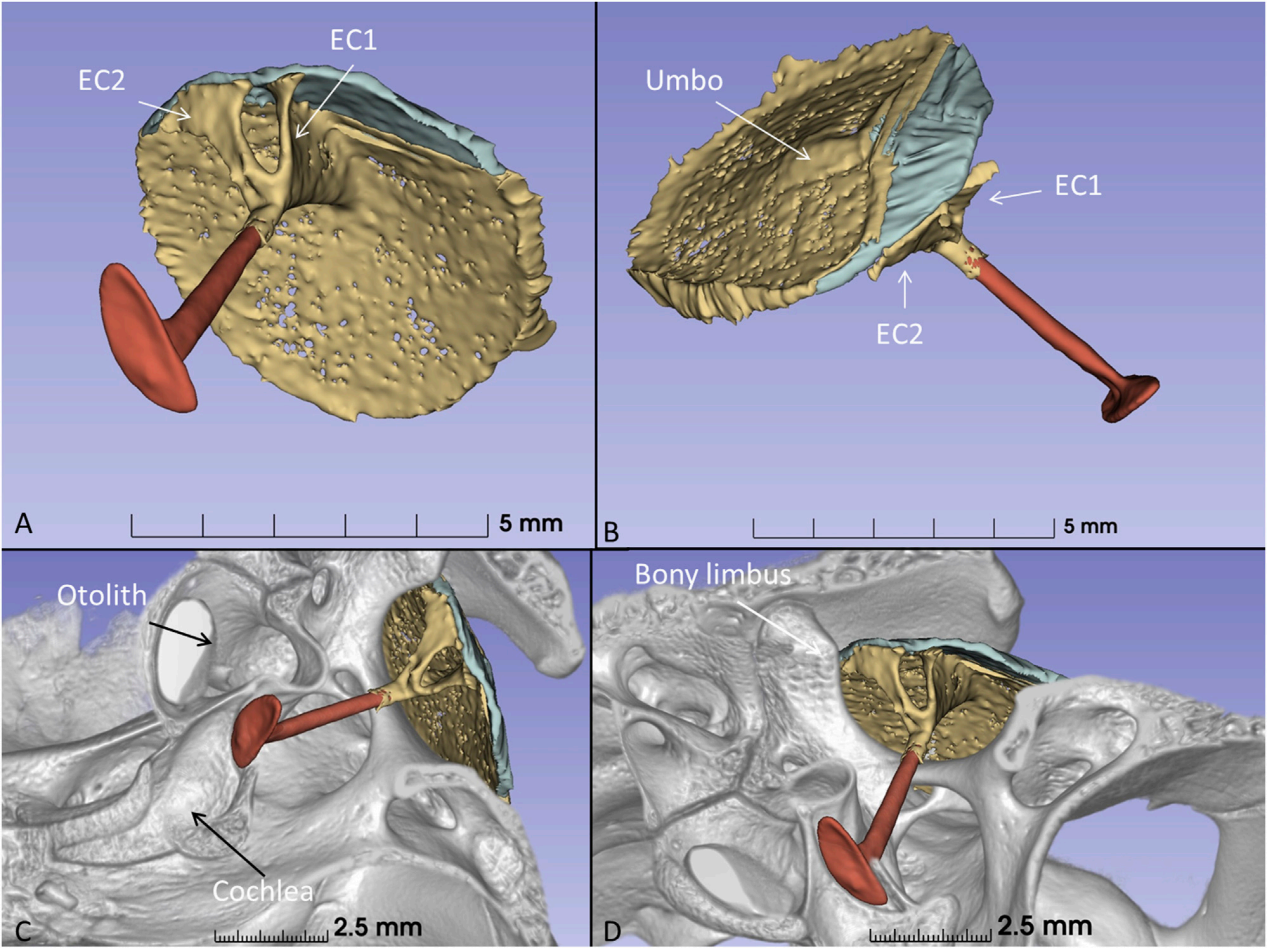
**(A)** µCT-3D segmentation of the right tympanic membrane in the seven-month-old *C. rhombifer* - inferior view). The anterior part is yellow, and the posterior part facing the superior earflap in the terminated crocodile is blue. The columella consists medially of a bony part with a stapes-like foot process in the oval window (red). It continues laterally into different components. One is attached to the ear drum and the umbo and runs posterior (EC1) while a second projects caudally (EC2) (yellow). **(B)** Supero-lateral view shows umbo and EC2 facing the tympanic annulus. **(C)** µCT 3D rendering and segmentations of the tympanic membrane and columella (inferomedial view). Its relationship to the cochlea and vestibule with otolith is seen. **(D)** EC2 adheres to the bony limbus.

The columella shaft was ossified medially, while the lateral part consisted of cartilage with components, referred to as the extracolumella (EC1 and EC2) (Figures 11A, B). EC1 connected to the central region of the tympanic membrane (the umbo), and extended posteriorly reaching the tympanic annulus. EC1 was triangular and sail-like, and connected posteriorly to the tensor tympani muscle. EC2 was spade-like and extended to the upper bony tympanic ring (Figures 11C, D). The tympanic membrane was arbitrarily separated into an anterior and posterior part that reached the inner surface of the superior earflap.

## Discussion

To our knowledge this is the first PCD-CT study of the live, spontaneously breathing crocodile using segmentation and 3D rendering. Used in combination with µCT in terminal, fixed animals, as a proxy for under water conditions, it may give us new information about tympanic aeration, ICE and provide additional information about crocodiles ability to use tympanic hearing under water and directional hearing.

According to current theories, tympanic ears seem to have evolved to be as efficient in water as in air to admit and relay sound vibrations (Mason et al., 2009; Vedurmudi et al., 2018). Crocodilians have large, subtle tympanic membranes and aerated tympani, uniquely protected behind plate-like earflaps, seemingly challenging sound to reach the ears for underwater hearing. Nonetheless, their hearing is excellent both in air and water including directional hearing (Higgs et al., 2002). The crocodilian earflaps are unique among reptiles and contain a sophisticated muscular machinery to regulate their function. Increased water pressure could move earflaps closer to the ear drum. In live crocodiles, the external ear canal is open anteriorly with a thin slit through the inferior earflap at a distance from the tympanic membrane, while submerged it closes deterring vibrations to reach the membrane. PCD-CT revealed the intriguing valvar structures opening and closing the meatus anteriorly.

**FIGURE 12:**
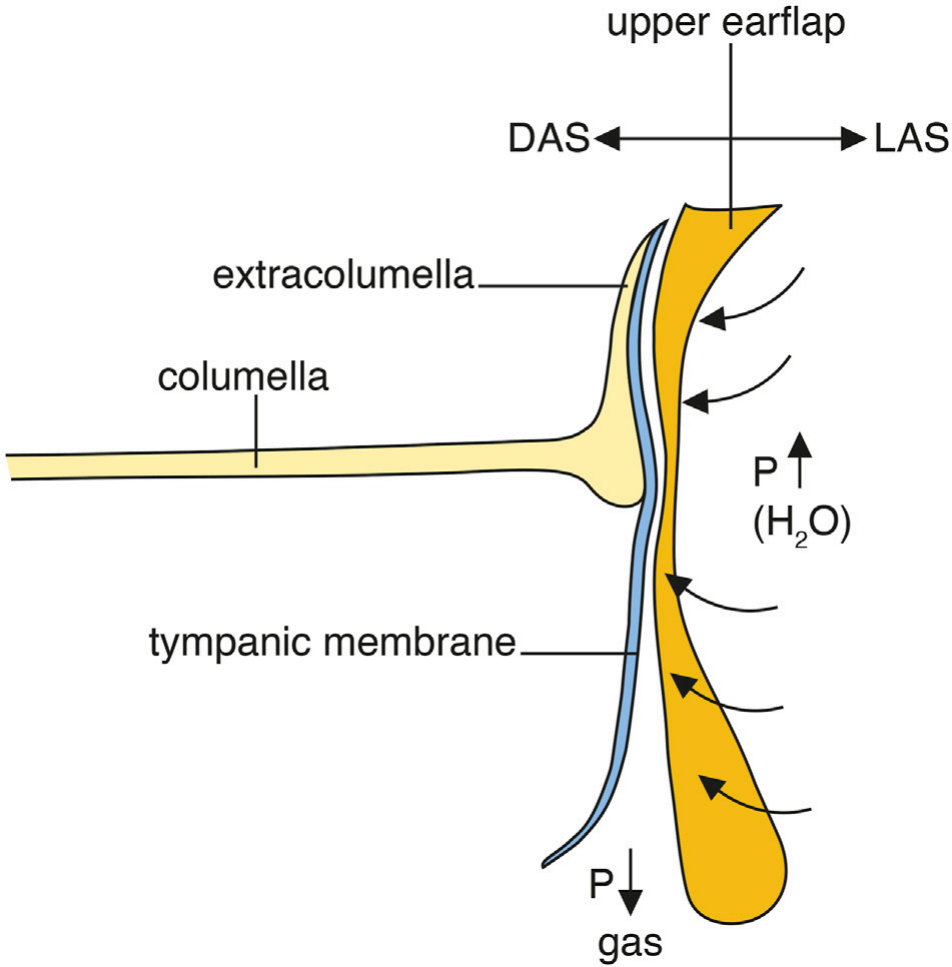
Hypothetical representation of tympanic underwater sound transmission and hearing in crocodiles. DAS; depressor auricular superior muscle. LAS; levator auricular superior muscle.

A potential route for sound to reach the middle ear under water is the superior earflap. Its involvement in sound transmission has generally been denied. Moreover, the superior earflap is described as separated from the posterior part of the tympanic membrane by a narrow MR (Shute and Bellairs, 1955). Under water, the volume of the MR may diminish due to gas absorption reducing pressure, not readily compensated by middle ear ventilation. A difference in pressure across the tympanic membrane together with the raised external water pressure could force the tympanic membrane and superior earflap together forming a direct pathway for vibrations to reach the inner ear. The earflap may thereby, besides serving as a protective shield, also function as an extra tympanic membrane under water (Figure 12).

### The role of the auricular muscles

PCD-CT and µCT revealed a different undulating surface structure of the superior earflap in closed and open MRs. It may depend on ventilation, volume pressure regulation and the forces acting on the earflap. It could also be due to the activity of the auricular muscles. The posterior region of the superior earflap contains an auricular plate of dense tissue similar to the tarsal plate of the mammalian eyelids (Shute and Bellairs, 1955). Its inner surface contained a specialized mucosa with gas regulating potential (Figures 9C, D). It is attached cranially as a hinge next to the squamosal allowing the plate to rotate through the action of muscles connected posteriorly behind the tympanic membrane and with the depressor muscle attached on its inner aspect (Killian, 1890). Microdissection confirmed the close relationship between the superior earflap muscles and the posterior canal wall, tympanic membrane and umbo (Supplementary Video S1). The prominent muscles act along a posterior sulcus narrowing the space between the posterior ear canal angle and the posterior region of the tympanic membrane overriding the extracolumella. In live animals these muscles may be actively involved in maintaining aeration of the posterior region of the meatal recess while in expired and fixed animals or underwater, a relaxation of these muscles could lead to a reduction/collapse of this part of the MR and fusion of the canal wall/earflap and the tympanic membrane. We postulate that these muscles can potentially influence the setting of the superior earflap and transfer vibrations directly to the tympanic membrane under water (Figure 12, Supplementary Figure S5). This would take advantage of the crocodile’s large tympanic membrane, its tension regulation and ICE for sound localization (Bierman and Carr, 2005; 2015; Köppl, 2009; Vergne et al., 2009; Dinets, 2013; Bierman et al., 2014; Hemmen et al., 2016; Vedurmudi et al., 2020; Tahara and Larsson, 2022; Young and Cramberg, 2024). It may operate together with their advanced cutaneous ISO-receptor system.

### Closure of meatal recess: A proxy for underwater hearing?

Closure of the ear canal and reduction of the distance between the tympanic membrane and earflap in terminated animals seems not to be caused by shrinkage. Fixatives were buffered and the osmotic pressure controlled reducing major swelling or shrinkage effects. In an anesthetized, terminated unfixed and directly frozen specimen the external orifice stayed open. Closure and earflap position are therefore likely modified by changes in muscular stiffness caused by the aldehyde fixative. If this represents a true proxy for underwater conditions may be argued. Shrinkage of tissue caused by fixation would conceivably result in a retraction/reduction of earflap tissue/thickness increasing the distance between the tympanic membrane and the earflap. Termination and aldehyde fixation seem to have an opposite effect. Nevertheless, the distance between the tympanic membrane (and extracolumella) and the superior earflap in the live crocodile was only 0.55 mm (Supplementary Figure S2, S3) and a direct acoustic transmission across the superior earflap seems plausible or even likely under some conditions. To fully understand the role of the superior earflap for underwater hearing it is necessary to apply further innovative *in vivo* investigations to prove whether it touches the tympanic membrane in submerged crocodiles. Conditions could also be disclosed by using endoscopic visualization via the anterior aperture of the ear canal. In a yet limited study, direct endoscopy of motion and coalescence of the superior earflap and the tympanic membrane in unfixed terminal crocodiles was implemented in our laboratory. This could further strengthen the view that the earflap transfers sound vibrations, either via internal muscular control or forces generated by increased ambient water pressure.

### Tympanic membrane and superior earflap surface interaction

An intriguing microscopic finding, that may lend further support of the superior earflap´s role to intermittently convey auditory transmission, was the lenient “mattress-like” structure of its interior surface wall (Figure 9E). This specialization could optimize physical adaptation to the tympanic membrane at underwater conditions. Thereby, the superior earflap may carefully respect the vulnerable membrane surface and simultaneously execute operative transmission of sound-induced vibrations.

### Aeration of the subtympanic space

The tympanic membrane overlies the floor of the meatal chamber (Montefeltro et al., 2015) and not only the middle ear opening as earlier described (Shute and Bellairs, 1955). An intriguing finding was the large, aerated space between the tympanic membrane and the shelf of bone surrounding the subtympanic foramen (Supplementary Figure S2, S3). The foramen was interpreted as a Helmholtz resonator essential for juvenile acoustic communication (David and Dufeau, 2015). One may speculate that this foramen may also serve as a dynamic aeration valve equalizing air pressure of the subtympanic space in the anterior region of the meatal chamber. It may ensure that the entire tympanic membrane is always operational in both air and underwater dwelling.

### Tympanic limbus and annulus

PCD-CT with µCT 3D rendering and segmentation disclosed the intriguing 3D outline of the extracolumella. Tympanic membrane and columella topography have previously been presented in embryonic and adult crocodiles by several authors (Parker, 1883; Goldby, 1925; Wever, 1978; Kundrát et al., 2009; Manley, 2016). A thorough analysis was undertaken recently in the American alligator (*Alligator mississippiensis)* (Young and Cramberg, 2024). The authors described the medial and caudal extracolumellae as extending along the margin of the tympanic membrane, fusing and forming an annular process. This process, connected to the tensor and depressor tympani muscles, may serve to modulate tympanic membrane tension. We found that the extracolumella cartilages diverged from the columnar shaft into the two perpendicular pillars, here called EC1 and EC2. EC1 resembled a sailcloth running from the umbo to the inner tympanic annulus and connecting to the tympanic tensor muscle. These conditions resemble human tensor tympani muscle insertion on the malleus shaft. The broad EC2 lay closely connected to the osseous tympanic limbus and tympanic annulus. This could provide an additional path for vibrations to reach the middle ear even though a specialized joint structure was not observed. The EC2 seems to stabilize the shaft and ear drum, while EC1 may regulate tympanic membrane tension. The tympanic annulus was robust and strongly developed attaching the tympanic membrane to the bony limbus. One may therefore speculate whether vibrations from the broad bony plate of the meatal chamber could also be involved in sound transfer to the tympanic membrane.

## Conclusion

From the present study using live PCD-CT and µCT we conclude that sound localization through tympanic hearing in submerged crocodiles is likely, whereby they may benefit from their inherent instruments together with central coding mechanisms. Crocodiles seem to have evolved the most ingenious biological modifications and innovations, impeccably fitted for bimodal airborne and submarine hearing.

## Data Availability

The original contributions presented in the study are included in the article/Supplementary Material, further inquiries can be directed to the corresponding author.

## Supplementary material

SUPPLEMENTARY FIGURE S1
**(A)** PCD-CT, 3D rendering, and segmentations of a live juvenile Cuban crocodile (*Crocodylus rhombifer*). Different algorithms and segmentations were used to show soft **(A)** and skeletal **(B)** tissue and different air-spaces **(C)**. **(D,E)** The tympanic air systems are internally coupled (IEC, red) and with paratympanic sinus systems (dorsal view). The crocodilian nose, larynx, and bronchial tree are visualized. The different arrangement of bronchi were analyzed in connection with their characteristic ancestral respiratory aerodynamics (Schachner et al., 2013).

SUPPLEMENTARY FIGURE S2 Serial frontal sections and 3D renderings demonstrate the physical relationship between the tympanic membrane and the inner wall of the superior earflap in the live spontaneously breathing Cuban crocodile. Red color denotes the aerated subtympanic spaces. Blue is the tympanic membrane and yellow is the extracolumella. At the posterior aspect of the tympanic membrane the distance to the superior earflap is 0.55 mm. Anteriorly at the subtympanic foramen the distance is 4.01 mm. red fiducials indicate min/max. aerated meatal recesses.

SUPPLEMENTARY FIGURE S3 Serial frontal sections and 3D renderings demonstrate the physical relationship between the tympanic membrane and the inner wall of the superior earflap in the live spontaneously breathing Cuban crocodile. Red color denotes the aerated subtympanic spaces. Blue is the tympanic membrane and yellow is the extracolumella. At the posterior aspect of the tympanic membrane the distance to the superior earflap is 0.55 mm. Anteriorly at the subtympanic foramen the distance is 4.01 mm. red fiducials indicate min/max. aerated meatal recesses.

SUPPLEMENTARY FIGURE S4
**(A)** µCT section of the left ear in the *Crocodylus moreletti*. Corresponding level of sectioning is shown at 3D rendering in **(B)**. The distance between the inner surface of the superior earflap (sf) and the tympanic membrane at umbo (arrows, red dots and staple) is 0.64 mm. SF; subtympanic foramen.

SUPPLEMENTARY FIGURE S5 µCT of the right meatal chamber **(A)** and corresponding soft tissue algorithm **(A)** in the expired *C. rhombifer*. The anterior orifice and mid-portion were opened to exclude accumulated meatal fluid during fixation The different frontal sections are shown in a-d. sf; superior earflap. if; inferior earflap. Non-aerated meatal recess is delineated in red. *; opened meatal recess.

SUPPLEMENTARY FIGURE S6 Artist concept of amphibian crocodile ear settings. Illustration by Karin Lodin.

SUPPLEMENTARY VIDEO S1 Anterior view displaying the neighboring connection between the superior earflap/posterior ear canal and the tympanic membrane in a left adult Cuviérs dwarf caiman (*Paleosuchus palpebrosus*). Aldehyde fixation and EDTA-decalcification.
