# Supplementary figures and images for: First photon-counting detector computed tomography in the living crocodile: a 3D-Imaging study with special reference to amphibious hearing
Source: Front Cell Dev Biol. 2024 Oct 23;12:1471983. doi: 10.3389/fcell.2024.1471983 (PMC11538886; doi:10.3389/fcell.2024.1471983)

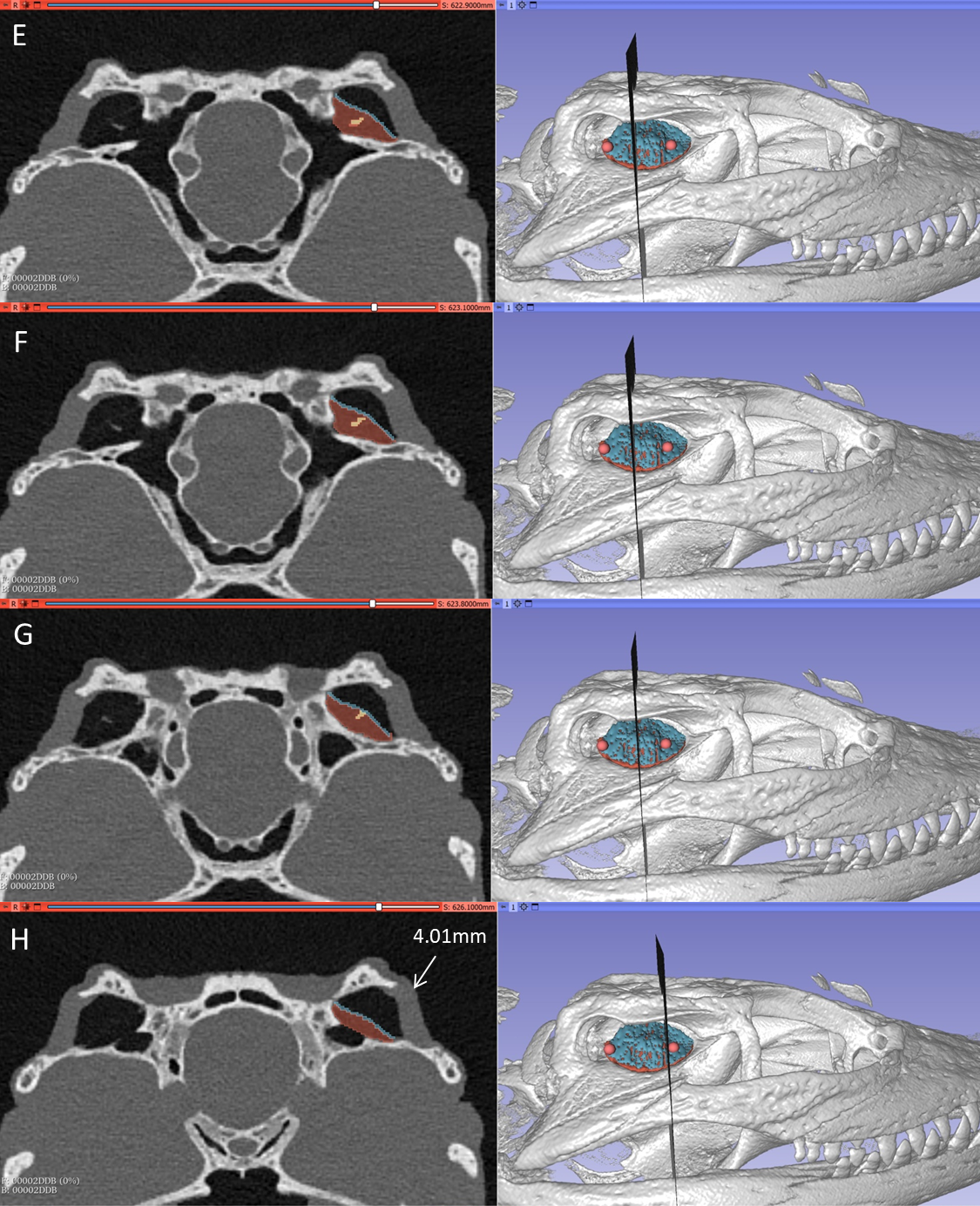

Supplement: Supplementary file 1 [file Image3.TIFF]

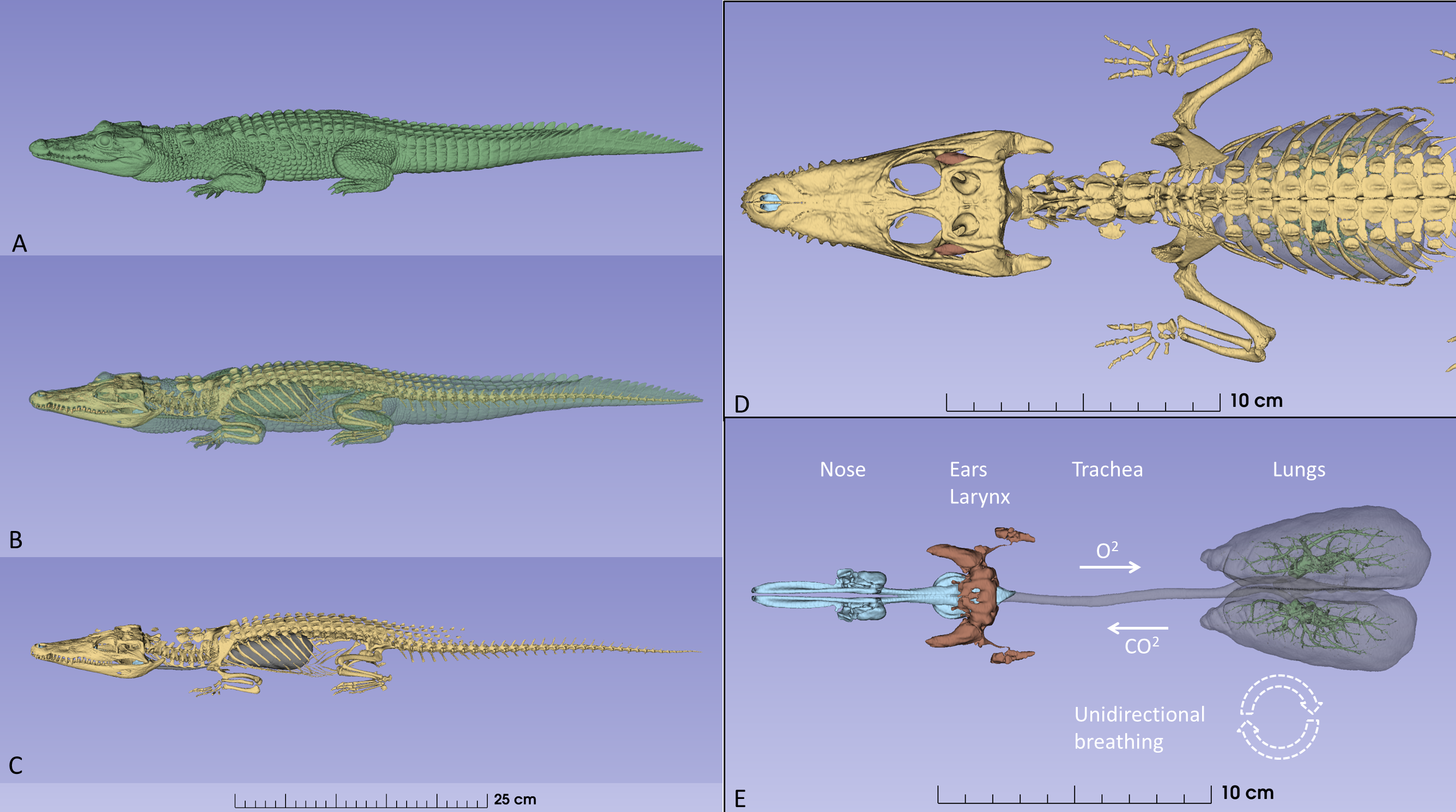

Supplement: Supplementary file 2 [file Image1.TIFF]

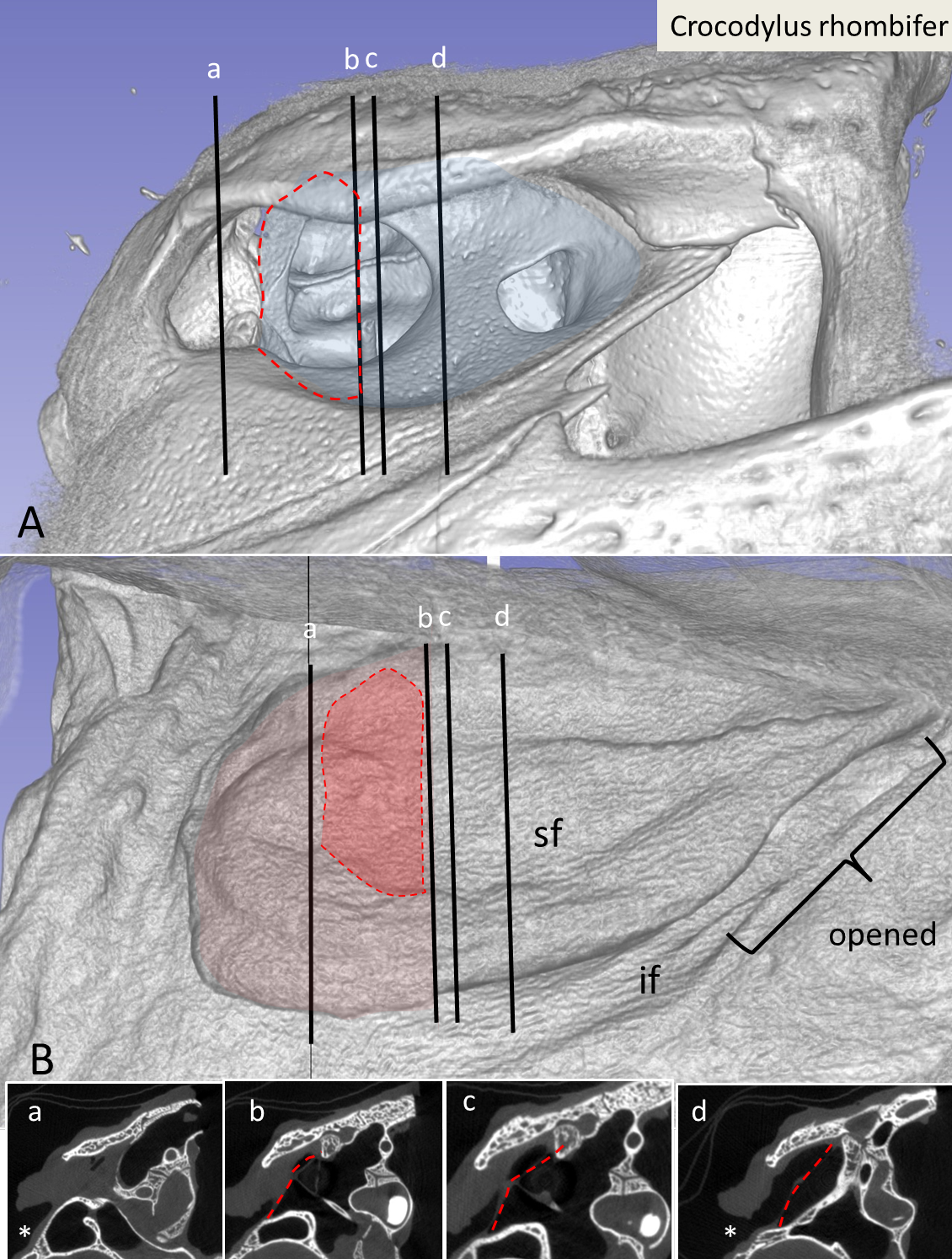

Supplement: Supplementary file 3 [file Image5.TIFF]

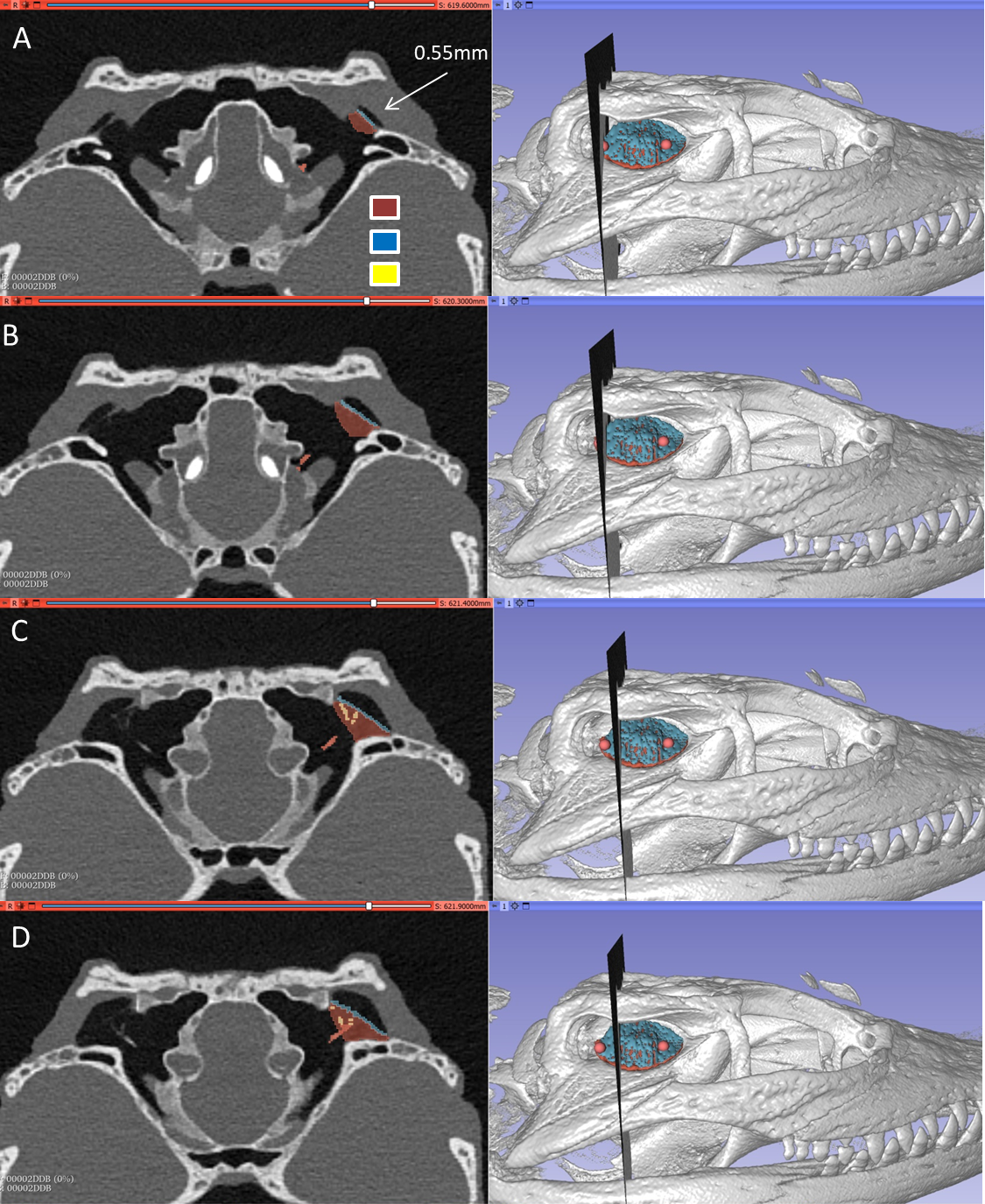

Supplement: Supplementary file 4 [file Image2.TIFF]

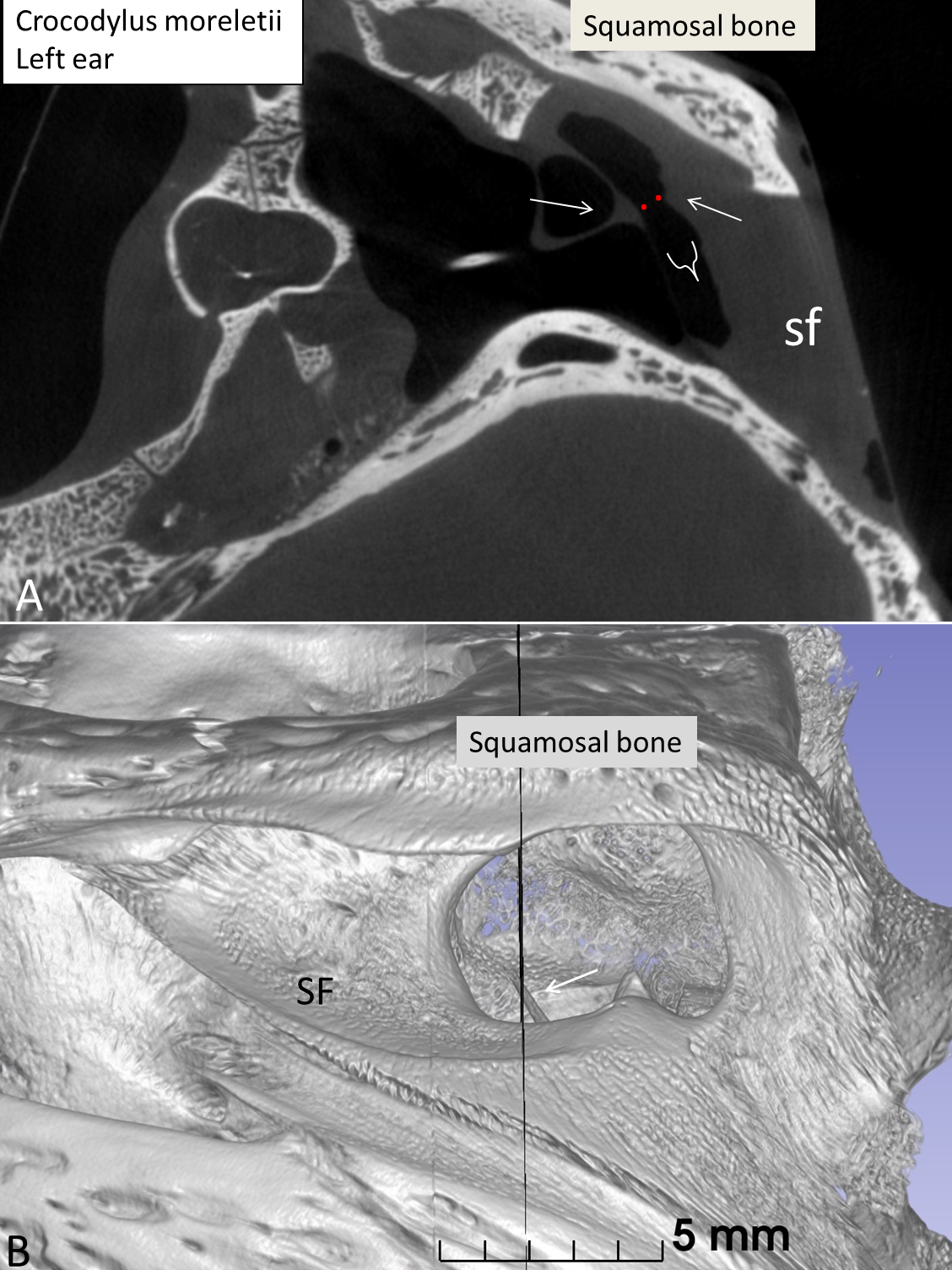

Supplement: Supplementary file 5 [file Image4.TIFF]

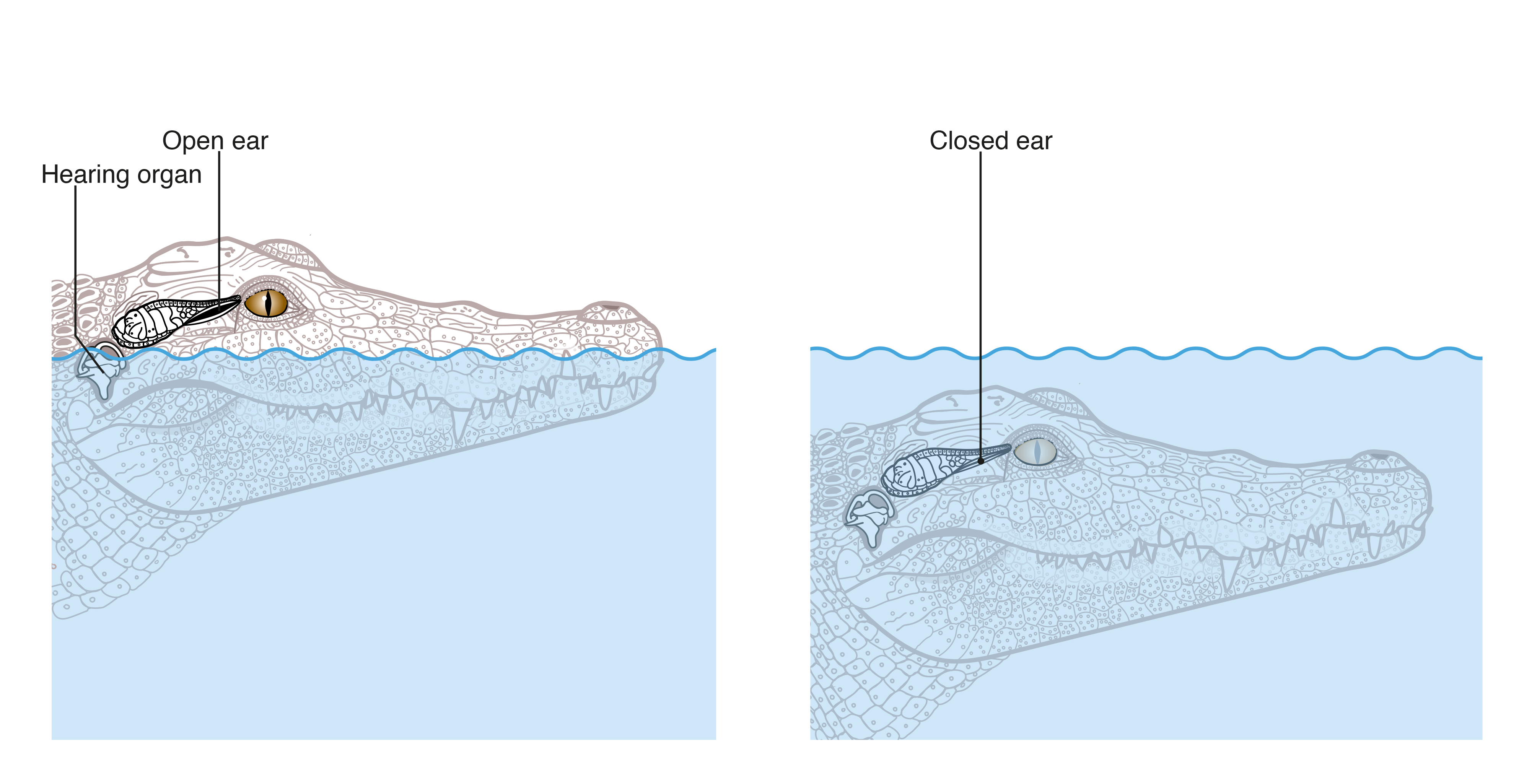

Supplement: Supplementary file 7 [file Image6.JPEG]
